# Non-Exchangeability of Running vs. Other Exercise in Their Association with Adiposity, and Its Implications for Public Health Recommendations

**DOI:** 10.1371/journal.pone.0036360

**Published:** 2012-07-13

**Authors:** Paul T. Williams

**Affiliations:** Life Sciences Division, Lawrence Berkeley National Laboratory, Berkeley, California, United States of America; Brigham and Women's Hospital and Harvard Medical School, United States of America

## Abstract

**Purpose:**

Current physical activity recommendations assume that different activities can be exchanged to produce the same weight-control benefits so long as total energy expended remains the same (exchangeability premise). To this end, they recommend calculating energy expenditure as the product of the time spent performing each activity and the activity's metabolic equivalents (MET), which may be summed to achieve target levels. The validity of the exchangeability premise was assessed using data from the National Runners' Health Study.

**Methods:**

Physical activity dose was compared to body mass index (BMI) and body circumferences in 33,374 runners who reported usual distance run and pace, and usual times spent running and other exercises per week. MET hours per day (METhr/d) from running was computed from: a) time and intensity, and b) reported distance run (1.02 MET•hours per km).

**Results:**

When computed from time and intensity, the declines (slope±SE) per METhr/d were significantly greater (P<10^−15^) for running than non-running exercise for BMI (slopes±SE, male: −0.12±0.00 vs. 0.00±0.00; female: −0.12±0.00 vs. −0.01±0.01 kg/m^2^ per METhr/d) and waist circumference (male: −0.28±0.01 vs. −0.07±0.01; female: −0. 31±0.01 vs. −0.05±0.01 cm per METhr/d). Reported METhr/d of running was 38% to 43% greater when calculated from time and intensity than distance. Moreover, the declines per METhr/d run were significantly greater when estimated from reported distance for BMI (males: −0.29±0.01; females: −0.27±0.01 kg/m^2^ per METhr/d) and waist circumference (males: −0.67±0.02; females: −0.69±0.02 cm per METhr/d) than when computed from time and intensity (cited above).

**Conclusion:**

The exchangeability premise was not supported for running vs. non-running exercise. Moreover, distance-based running prescriptions may provide better weight control than time-based prescriptions for running or other activities. Additional longitudinal studies and randomized clinical trials are required to verify these results prospectively.

## Introduction

Current physical activity recommendations [1]–[6] assume that different moderately and vigorously intense physical activities can be exchanged to produce the same health benefits so long as total energy expended remains the same (exchangeability premise). To this end, they recommend calculating energy expenditure as the product of the time spent performing each activity and the activity's metabolic equivalents (MET, representing their X-fold increase in energy expenditure relative to sitting at rest, 1 MET = 3.5 ml O_2_•kg^−1^•min^−1^ [7]), which may then be summed and compared to target levels. This approach is pragmatically useful, in that it provides flexibility for individuals to tailor an exercise program in accordance to their own preferences [2]. There is, however, little direct evidence that the sum total of these time-based MET calculations across multiple physical activities provides the best metric for exercise prescription.

**Figure 1:**
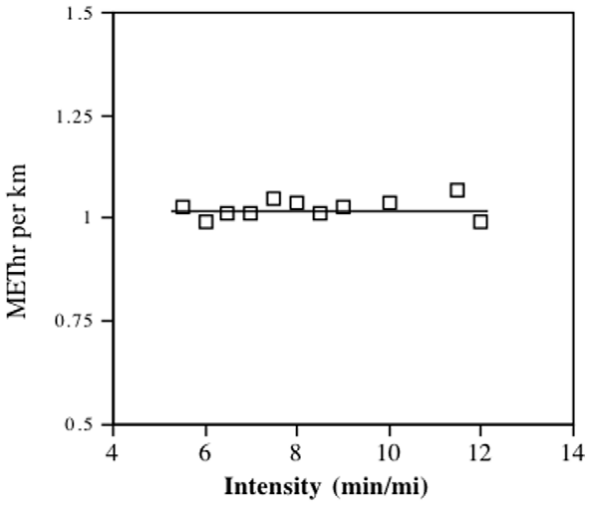
Metabolic equivalent hours per day (METhr/d) per km/d run from the published compendium values by Ainsworth et al [7].

The exchangeability premise probably derives from two factors: 1) many epidemiological studies have had limited statistical power to assess the effects of specific physical activities on morbidity and mortality, requiring that different activities be pooled using a common metric; and 2) the energy balance perspective in obesity research, i.e., weight gain or loss is primarily the result of energy excess or deficit, irrespective of the mode or the intensity of the exercise that contributes to this balance [5], [8]. The National Runners' Health Study is unique among all large epidemiological studies in targeting a specific physical activity for study [9], [10]. It has shown that baseline running distance, in particular, reduced weight gain over time [11] when other cohort studies have had difficulty showing physical activity affects body weight prospectively [12]. Although the success of the National Runners' Health Study is probably due in part to the statistical power provided by the cohort's large sample size and broad activity range, it is also possible that the exchangeability premise is invalid and that the health benefits of energy expended by running are greater than for energy expended by other exercise. In addition, running may be more accurately reported than other physical activities because it can be calculated as a function of distance only (i.e., independent of running intensity, see Figure 1), as opposed to being calculated as the product of time and intensity. If the latter is true, then this could have important public health implications. Specifically, it could mean that public health targets based on running distance might be more effectively implemented than physical activity targets based on time and intensity.

Re-survey of the National Runners' Health Study in 2006 included survey questions on the usual time spent running, walking, cycling, swimming, and other exercises, in addition to usual distance run per week and the time required to run one mile. The runners reported a variety of activities in addition to running. Those activities requiring <3 METs were classified as light intensity, 3 to 6 METs as moderate intensity, and >6 METs as vigorous intensity [1], [7]. Running is a vigorous activity [7]. The MET hours per day for each intensity was calculated as sum total of the average hours spent per day on each activity times its MET value [3], [7]. These were used to test the exchangeability premise in terms of exercise's association with excess body weight, i.e., whether the relationships of BMI, body circumferences, and total and abdominal obesity to METhr/d of exercise were the same for running vs. all other exercise, and for running vs. all other vigorous exercise. In addition, we tested whether differences in the relationship were attributable to the superiority of the distance-based calculation of METhr/d run over its time and intensity-based calculation, as used for other activities. These results suggest possible improvements in the prescription of exercise targets, both in defining targeted goals and in the types of exercise prescribed.

## Results

The men ran an average of 4.00 km/d in 0.49 hours, women an average of 3.48 km/d in 0.46 hours. Estimated METhr/day of running activity was 38% greater when calculated from self-reported time (hours) and intensity (pace) than from usual distance in men (mean±SD: 5.58±5.39 vs. 4.02±3.17 METhr/d), and 43% greater in women (4.96±5.29 vs. 3.48±3.04 METhr/d). On average, when calculated from usual distance (Running_Distance_), running represented 54.8±33.3% of the total METhr/d expended by exercise in men, and 45.0±32.1% in women. When calculated from time and intensity (Running_Time_), running represented 58.3±33.0% and 49.0±33.0% of the total in men and women, respectively. Running_Distance_ and Running_Time_ were correlated r = 0.62 in men and r = 0.64 in women. Table 1 presents the characteristics of the sample by METhr/d run. The longer-distance runners tended to be younger, smoke less, and eat less meat and more fruit.

**Table 1:** Sample characteristics (±SD) by quartiles of METhr/d from self-reported distance run.

	Quartiles of METhr/d from self-reported distance run			
	1^st^ quartile	2^nd^ quartile	3^rd^ quartile	4^th^ quartile
Age (years)				
Male	55.62±12.15	55.31±11.14	54.66±10.52	53.12±10.36
Female	48.81±11.76	46.99±10.71	47.29±10.13	46.84±10.00
Education (years)				
Male	16.73±2.44	16.94±2.40	16.82±2.42	16.71±2.64
Female	16.26±2.37	16.43±2.23	16.45±2.22	16.28±2.42
Smokers (%)				
Male	1.68	1.20	1.13	0.72
Female	2.40	1.46	1.14	1.03
Meat (servings/day)				
Male	0.49±0.48	0.49±0.51	0.45±0.43	0.42±0.41
Female	0.34±0.37	0.32±0.33	0.31±0.32	0.27±0.66
Fruit (pieces/day)				
Male	1.39±1.61	1.46±1.30	1.47±1.12	1.71±3.12
Female	1.52±1.65	1.49±1.09	1.60±1.07	1.74±2.07
Alcohol (g/day)				
Male	11.30±14.66	11.67±14.25	11.35±15.81	10.72±14.81
Female	6.50±8.88	7.09±9.03	7.61±9.85	7.32±10.69
BMI (kg/memphtype				
Male	26.05±3.76	25.28±3.10	24.67±2.77	23.70±2.60
Female	23.47±4.01	22.55±3.01	21.95±2.54	21.16±2.35
Waistcircumference (cm)				
Male	89.33±8.43	87.58±6.91	86.08±6.42	83.67±6.08
Female	75.55±9.75	73.42±7.93	71.69±7.12	69.68±6.57
Hipcircumference (cm)				
Female	95.77±9.22	93.86±7.79	92.29±6.96	90.06±6.67
Chestcircumference (cm)				
Male	104.51±9.21	103.43±8.12	102.69±7.89	100.70±7.70
Female	91.10±7.07	89.69±5.52	88.91±5.28	87.67±5.14
Non-healthyweight (%)				
Male	57.95	50.46	41.36	26.05
Female	25.30	16.58	10.34	5.23
Obese (%)				
Male	12.38	6.68	3.90	2.39
Female	6.75	2.44	1.31	0.70
Abdominalobesity (%)				
Male	6.10	2.54	1.52	0.52
Female	11.13	5.22	2.97	1.42
Otherexercise METhr/d				
Male	5.02±5.41	3.90±5.85	3.47±4.26	3.65±4.57
Female	5.26±5.10	4.60±4.67	4.28±4.56	4.70±5.12

### BMI and body circumferences

Consistent with our previously published reports [9], [10], Tables 1, 2, 3 show that BMI and circumferences of the waist, hip and chest were inversely related to METhr/d run in both men and women. When calculated from reported distance, METhr/d run had a substantially stronger relationship to BMI and body composition than METhr/d from other exercise. For example, BMI declined 0.29 kg/m^2^ per METhr/d run but only 0.02 kg/m^2^ per METhr/d from other exercise, a 19.3-fold difference. In women, the difference was 13.9-fold. Compared to other exercise, the estimated effects per METhr/d run in men and women were 6.8- and 9.5-fold greater for waist circumference, respectively, and 9.0-fold greater for women's hip circumference.

**Table 2:** Cross-sectional regression slopes (±SE) of BMI and body circumferences (dependent variables) versus MET hours per day of running and other physical activities (independent variables) in males.

	BMI (kg/m^2^)	Waist circumference (cm)	Chest circumference (cm)
**Distance-based estimate**			
Running_Distance_ coefficient	-0.29±0.01	-0.67±0.02	-0.50±0.02
Otherexercise coefficient	-0.02±0.00‡	-0.10±0.01§	0.06±0.01§
*Difference: Running_Distance._ -other exercise coefficients*	-0.28±0.01	-0.57±0.02	-0.56±0.02
Running_Distance_ coefficient	-0.29±0.01	-0.66±0.02	-0.50±0.02
Othervigorous exercise coefficient	-0.03±0.01§	-0.13±0.01§	0.02±0.02
Light &moderate exercise coefficient	0.02±0.01	-0.01±0.02	0.18±0.03§
*Difference: Running_Distance._ -other vigorous coefficients*	-0.27±0.01	-0.53±0.02	-0.52±0.03
**Time-basedestimate**			
Running_ Time_ coefficient	-0.12±0.00	-0.28±0.01	-0.20±0.01
Otherexercise coefficient	0.00±0.00	-0.07±0.01§	0.08±0.01§
*Difference: Running_Time_ -other exercise coefficients*	-0.12±0.01	-0.21±0.01	-0.28±0.02
Running_ Time_ coefficient	-0.12±0.00	-0.28±0.01	-0.20±0.01
Othervigorous exercise coefficient	-0.02±0.01‡	-0.11±0.01§	0.04±0.02*
Light &moderate exercise coefficient	0.04±0.01§	0.05±0.02*	0.22±0.03§
*Difference: Running_Time_ -othervigorous coefficients*	-0.10±0.01	-0.17±0.02	-0.24±0.02

Adjusted for age, education, current smoking status, and intakes of meat, fruit, and alcohol. Significance of the regression coefficients and their differences coded*P<0.05; †P<0.01; ‡P<0.001; §P<0.0001; P<10^-15^, in the model: Dependent variable = intercept+αRunning_Distance_+βOther exercise+covariates, or Dependent variable = intercept+αRunning_Time_+βOther exercise+covariates.

**Table 3:** Cross-sectional regression slopes (±SE) of BMI and body circumferences (dependent variables) versus METhours per day of running and other physical activities (independent variables) in females.

	BMI (kg/m^2^)	Waist Circumference (cm)	Chest Circumference (cm)	Hip Circumference (cm)
**Distance-based estimate**				
Running_Distance_ coefficient	-0.27±0.01	-0.69±0.02	-0.39±0.02	-0.68±0.02
Otherexercise coefficient	-0.02±0.00§	-0.07±0.01§	-0.01±0.01	-0.08±0.01§
*Difference: Running_Distance_ -other exercise coefficients*	-0.25±0.01	-0.61±0.03	-0.38±0.02	-0.61±0.03
Running_Distance_ coefficient	-0.27±0.01	-0.68±0.02	-0.39±0.02	-0.68±0.02
Othervigorous exercise coefficient	-0.03±0.01§	-0.09±0.02§	-0.03±0.01*	-0.09±0.02§
Light &moderate exercise coefficient	0.00±0.01	-0.03±0.03	0.03±0.02	-0.04±0.03
*Difference: Running_Distance_-other vigorous coefficients*	-0.24±0.01	-0.59±0.03	-0.36±0.02	-0.59±0.03
**Time-basedestimate**				
Running_ Time_ coefficient	-0.12±0.00	-0.31±0.01	-0.19±0.01	-0.32±0.01
Otherexercise coefficient	-0.01±0.00†	-0.05±0.01§	0.00±0.01	-0.06±0.01§
*Difference: Running_Time_ -other exercise coefficients*	-0.11±0.01	-0.26±0.02	-0.19±0.01	-0.26±0.02
Running_ Time_ coefficient	-0.12±0.00	-0.31±0.01	-0.19±0.01	-0.32±0.01
Othervigorous exercise coefficient	-0.03±0.01§	-0.09±0.02§	-0.03±0.01*	-0.09±0.02§
Light &moderate exercise coefficient	0.01±0.01	0.02±0.03	0.05±0.02†	0.01±0.03
*Difference: Running_Time_-other vigorous coefficients*	-0.10±0.01	-0.22±0.02	-0.16±0.02	-0.23±0.02

Adjusted for age, education, current smoking status, and intakes of meat, fruit, and alcohol. Significance of the regression coefficients andtheir differences coded*P<0.05; †P<0.01; ‡P<0.001; §P<0.0001; P<10^-15^, in the model: Dependent variable = intercept+αRunning_Distance_+βOther exercise+ covariates, or Dependent variable = intercept+αRunning_Time_+βOther exercise + covariates.

Tables 2 and 3 show that the decline in BMI and body circumferences per MET*hr/d run were over twice as great when calculated from reported km/day than when calculated from time and intensity. For example, men's BMI declined (slope±SE) −0.29±0.01 kg/m^2^ per METhr/d run when calculated from reported distance but only −0.12±0.00 kg/m^2^ per METhr/d for its traditional calculation from time, nearly a 2.4-fold difference. In women, the corresponding comparison was a 2.2–fold difference. Similarly, for body circumferences, the men and women's declines per METhr/d run were between 2- and 2.5-fold greater when calculated from reported distance than from reported time.

The preceding analyses of running vs. other exercise were based on METhr/d for running from usual distance run, and METhr/d for other exercise from time spent exercising. With respect to evaluating their physiological effect on body weight, it may make more sense to estimate METhr/d for running using the same metric as used for other exercise (time). The tables show that when all activities, both running and non-running, were estimated from time and intensity, the associations were significantly stronger (P<10^−15^) for running than non-running exercise. Moreover, the difference is not simply attributable to running being a vigorous activity, and other activities including moderate and light activities. In both sexes, and for all reported body measurements, MET*hr/d from running was more strongly related to adiposity than MET*hr/d from other vigorous exercise (P<10^−15^), in contradiction to the exchangeability premise. Light to moderate-intensity exercise showed only modest associations with the runners' BMI and body circumferences.

Table 4 displays the multiple linear regression analyses of the declines in BMI and body circumference per METhr/d run when the distance- and time-based calculations are both included simultaneously in the model. Although significant, the time-based calculation produced little additional improvement in the model over the distance calculation, while the distance calculation remained wildly significant even when adjusted for the time-based calculation (all P<10^−15^). In addition, Table 4 shows that the coefficient for distance-based METhr/d run is, in every case, significantly greater than the coefficient for time-based METhr/d run. The significantly greater effect of Running_Distance_ than other exercise on BMI and body circumferences persisted when adjusted for Running_Time_.

**Table 4:** Cross-sectional regression slopes (±SE) of BMI and body circumference measurements (dependent variables) versus METhours per day of running and other physical activities (independent variables).

	BMI (kg/m^2^)	Waist Circumference(cm)	Chest Circumference(cm)	Hip Circumference(cm)
**Males**				
Regression coefficients (METhr/d)				
Running_Distance_	-0.27±0.01	-0.61±0.02	-0.47±0.03	
Running_Time_	-0.03±0.01§	-0.05±0.01§	-0.03±0.02	
Otherexercise	-0.01±0.00†	-0.10±0.01§	0.06±0.01§	
Differencesbetween regression coefficients				
Running_Distance_ - Running_Time_	-0.24±0.01	-0.56±0.03	-0.44±0.04	
Runningemphtype	-0.25±0.01	-0.52±0.02	-0.53±0.03	
Running_Time._ - Other Exercise	-0.01±0.01	0.04±0.02†	-0.09±0.02§	
**Females**				
Regression coefficients (METhr/d)				
Running_Distance_	-0.22±0.01	-0.57±0.03	-0.29±0.02	-0.55±0.03
Running_Time_	-0.04±0.01§	-0.11±0.02§	-0.08±0.01§	-0.12±0.02§
Otherexercise	-0.02±0.00‡	-0.07±0.01§	-0.01±0.01	-0.07±0.01§
Differencesbetween regression coefficients				
Running_Distance_ - Running_Time_	-0.18±0.01	-0.46±0.04	-0.21±0.03	-0.42±0.04
Running_Distance._ - Other Exercise	-0.20±0.01	-0.50±0.03	-0.29±0.02	-0.48±0.03
Running_Time_ - Other Exercise	-0.03±0.01‡	-0.04±0.02	-0.07±0.02§	-0.05±0.02

Adjusted for age, education, current smoking status, and intakes of meat, fruit, and alcohol. Significance of the regression coefficients andtheir differences coded*P<0.05; †P<0.01; ‡P<0.001; §P<0.0001; P<10^-15^ in the model: Dependent variable = intercept+αRunning_Distance._+ βRunning_Time._+ γOtherexercise+covariates. Abbreviations: BMI, body mass index; MET, metabolic equivalents of energy expenditure; Running_Distance_, metabolic equivalent hr/d from running as estimated from self-reported distance, Running_Time_, metabolic equivalent hr/d from running as estimated from self-reported duration.

### Total and abdominal obesity in runners

Table 5 shows that distance-derived and time-derived METhr/d run were both inversely related to the prevalence of total and abdominal obesity in both men and women (P<10^−15^). Without exception, the estimated effects of running on adiposity were greater than the estimated effects of other exercise. The reductions in odds were between 29% and 125% greater per METhr/d run when computed from distance than when computed from time spent running.

**Table 5:** Odds ratio (95% confidenceinterval) for obesity and abdominal obesity versus METhours per day ofrunning and other physical activities.

	Obesity (BMI≥30)		AbdominalObesity	
	males	females	males	females
Distance-based estimate				
Running_Distance_	0.77	0.69	0.69	0.73
	(0.75,0.79)	(0.65,0.73)	(0.66,0.73)	(0.71,0.76)
Otherexercise	0.99	0.97‡	0.98	1.00
	(0.98,1.01)	(0.95,0.99)	(0.96,1.00)	(0.98,1.01)
*Running_Distance_ vs. other exercise*	*P<10^-15^*	*P<10^-15^*	*P<10^-15^*	*P<10^-15^*
Running_Distance_	0.77	0.69	0.69	0.73
	(0.75,0.79)	(0.65,0.73)	(0.66,0.73)	(0.71,0.76)
Othervigorous exercise	0.98	0.94‡	0.92§	0.98
	(0.96,1.00)	(0.91,0.97)	(0.89,0.96)	(0.96,1.00)
Light &moderate exercise	1.01	0.95†	1.01	1.00
	(0.99,1.03)	(0.92,0.99)	(0.98,1.05)	(0.97,1.03)
*Running_Distance_ vs. other vigorous*	*P<10^-15^*	*P<10^-15^*	*P<10^-15^*	*P<10^-15^*
Time-basedestimate				
Running_Time_	0.85	0.76	0.78	0.82
	(0.83,0.87)	(0.73,0.79)	(0.75,0.81)	(0.80,0.84)
Otherexercise	1.00	0.97†	0.98	1.00
	(0.99,1.01)	(0.95,0.99)	(0.96,1.00)	(0.99,1.01)
Running_Time_ *vs. other exercise*	*P<10^-15^*	*P<10^-15^*	*P = 10^-15^*	*P<10^-15^*
Running_Time_	0.85	0.76	0.78	0.82
	(0.83,0.87)	(0.73,0.79)	(0.75,0.81)	(0.80,0.84)
Othervigorous exercise	0.99*	0.94‡	0.92§	0.98
	(0.97,1.00)	(0.91,0.98)	(0.89,0.96)	(0.96,1.01)
Light &moderate exercise	1.02	0.95*	1.02	1.00
	(1.00,1.04)	(0.92,1.00)	(0.99,1.06)	(0.98,1.03)
Running_Time._ *vs. other vigorous*	*P<10^-15^*	*P = 2x10^-15^*	*P = 5.7x10^-10^*	*P<10^-15^*

Adjusted for age, education, current smoking status, and intakes of meat, fruit, and alcohol. Significance of the regression coefficients coded *P<0.05; †P<0.01;‡P<0.001; §P<0.0001; P<10^-15^, in the model: ln(p/(1-p)) = intercept+αRunning_Distance_+βOther exercise + covariates, orln(p/(1-p)) = intercept+αRunning_Time_ +β Other exercise + covariates. Abbreviations: BMI, body mass index; MET, metabolic equivalents of energy expenditure; Running_Distance_, metabolic equivalent hr/d from running as estimated from self-reported distance, Running_Time_, metabolic equivalent hr/d from running as estimated from self-reported duration.

**Figure 2:**
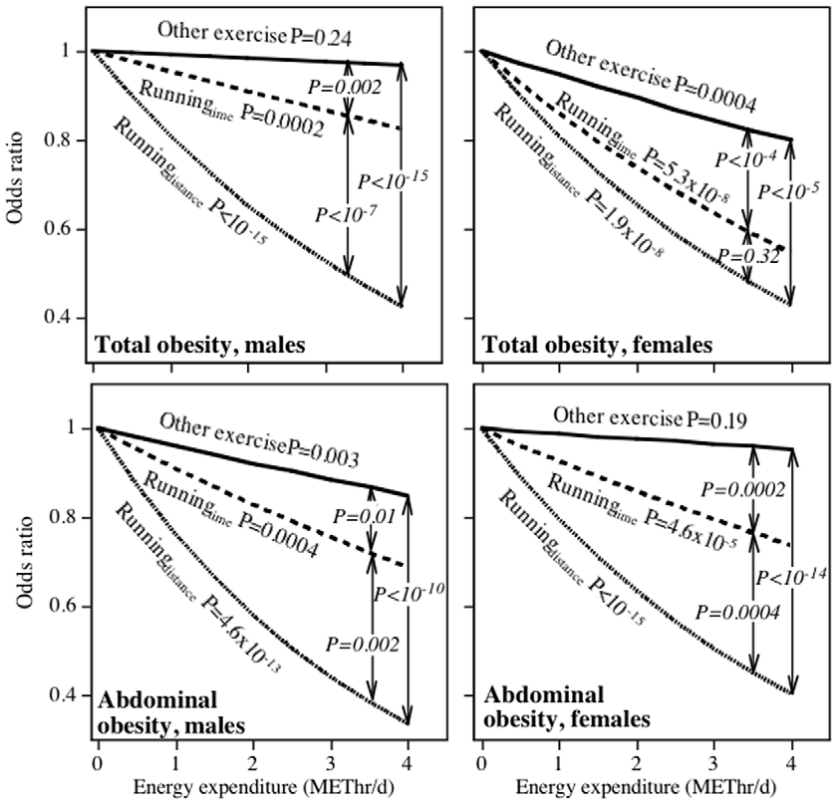
Odds reduction in obesity (BMI≥30 kg/m^2^), and abdominal obesity per METhr/d energy expenditure. Significance levels presented for α = β, α = γ and β = γ in the model: ln(p/(1-p)) = intercept+αRunning_Distance_+βRunning_Time_ +γOther exercise +covariates.

Figure 2 displays the odds reductions for the independent effects of distance-based and time-based METhr/d run on total and abdominal obesity from their logistic regression analyses. For example, in men, the odds for obesity decreased 19.26% per METhr/d run when calculated from distance and adjusted for time-based METhr/d, but only 4.71% per METhr/d run when calculated from time spent running and adjusted for distance. Although both effects are statistically significant, the large sample size provides considerable power to assign statistical significance to even small effects. The significant independent contribution of the time-based calculation could imply that there is a duration (time) effect that is not captured by the distance calculation, that some people are better at estimating their METhr/d running as time, or both. Of greater interest is the fact that the estimated effect METhr/d run on obesity was significantly greater for the distance-based calculation than the time-based calculation in men, albeit not in women. The remaining graphs show that the independent effect per distance-based METhr/d run was significantly greater than the corresponding time-based estimate for abdominal obesity in men (23.11 vs. 8.89%) and women (19.82 vs. 10.73%).

## Discussion

The National Institutes of Medicine recommend 45 to 60 minutes per day of moderate to vigorous physical activity to prevent transition to overweight or obesity, and 60–90 min of moderate-intensity physical activity per day to prevent weight gain after substantial weight loss [5]. The dietary guidelines for Americans 2010 [6] state “To achieve and maintain a healthy body weight, adults should do the equivalent of 150 minutes of moderate-intensity aerobic activity each week…Some adults will need a higher level of physical activity than others to achieve and maintain a healthy body weight. Some may need more than the equivalent of 300 minutes per week of moderate-intensity activity.” By equivalent, “one minute of vigorous-intensity physical activity counts as two minutes of moderate-intensity physical activity toward meeting the recommendations”.

The exchangeability premise is a central tenant of these recommendations. Specifically, they assume that the contribution of any specific physical activity to body weight is limited to its contribution to total energy expenditure. In part, it may be a reflection of the inability of the doubly labeled water to distinguish energy expended by different activities [13]. Almost all prospective epidemiological studies reported the relationship of body weight to total energy expenditure without regard to intensity [12], even though intensity data were obviously available. Although this could reflect the absence of any significant results for intensity, it could also indicate an entrenchment of the exchangeability premise, such that its alternatives are not considered. For example, a recent comprehensive review [12] of the causes of weight gain, introduced physical activity with the statement: “… the mechanistic significance of physical activity or inactivity as a determinant of weight gain relates to the totality of physical activity, rather than to domain-specific components such as transportation, domestic life, leisure and occupation. Similarly, with respect to the thermodynamics of energy balance, specific physical activity exposures such as frequency, intensity and duration of activity are important only in their combined contribution to total PAEE [physical activity energy expenditure] or TEE [total energy expenditure]”.

Generally, the various public health recommendations cite no evidence for the validity of the exchangeability premise. In fact, the preponderance of the evidence appears to suggest that vigorous exercise has greater effect on reducing cardiovascular disease risk in men [14]. Cross-sectionally, vigorous physical activity has been associated with lower total and abdominal obesity [15] independent of total energy expenditure. The fact that epidemiological studies show that higher cumulative METmin/d of physical activity are associated with lower body weight and lower disease risk does not validate the exchangeability premise. Higher total energy expenditure is associated with higher doses of vigorous exercise, of which running is among the more common vigorous activities [16].

The greater effects of running vs. other exercise (Tables 2,3,5) would appear to negate the simplicity of the exchangeability premise. Although obesity arises from positive net energy balance between intake and expenditure, several mechanisms have been identified by which exercise may affect body weight beyond the energy required to produce the activity. Exercise may affect body weight in a positive manner through improved eating behaviors [17], i.e., improved homeostatic appetite control through hunger–satiety mechanisms [18], reduced binge consumption in response to negative emotions [19], and better self-control [20]. The improvement in appetite control following increased exercise is reported to produce a negative energy balance in some [18], albeit not all [21] individuals. In this regard, obesity is also a neurobehavioral disorder, with the coupling of energy intake and expenditure being tightly regulated by hypothalamic factors, the hypothalamus mediating exercise-induced appetite suppression, and exercise sensitizing the hypothalamus to leptin and insulin [22]. Postprandial increases in the purported satiety hormones occur after exercise [23], [24]. Other effects of exercise that may affect obesity beyond energy expenditure include: 1) increased brain BDNF levels, 2) decreasing plasma and pancreatic β-cell content of IL-6 and TNF-α, 3) increasing parasympathetic tone, and 4) anti-inflammatory effects of exercise [22]. Regular exercisers also demonstrate a greater ability to compensate for overeating by consuming fewer calories at subsequent meals [25]. Some of these effects may explain why physical activity is consistently associated with successful long-term weight control [26], [27]. In fact, most of the genetic associations with obesity discovered to date appear to be related to food intake, satiety, and hunger rather than energy balance [28]. Exercise is also reported to increase post-exercise energy expenditure [29], and resting metabolic rate [30], [31] in accordance with increased fat-free body mass.

The compendium of physical activities and various public health guidelines specifically define energy expenditure as the product of the duration (time) and the MET values of the activity [7]. Among runners, running may be more naturally measured by distance than time. Figure 1 shows that the MET values for different running intensities are a simple function of distance run. However, energy expended by other physical activities is not so easily quantified, requiring proof that the different relationships of body weight to running and non-running exercise were not simply explained by their different energy calculations. Tables 2, 3 and 5 showed that the stronger relationships persisted when all energy expenditures were calculated using the same metric (time). These analyses also showed the superiority of the distance-based calculation of METhr/d over its time-based calculation. Presumably, errors and biases affecting the time-based calculation of energy expenditure by running also affect its calculation for other physical activities, suggesting that time-based calculations used by virtually all epidemiological studies may substantially underestimate the health benefits of physical activity. Our use of a distance-based calculation for estimating energy expenditure may explain in part our ability to assess strongly significant associations between physical activity and body weight and circumferences in the National Runners' Health Study [11].

Our analyses suggest that time-based estimates overestimated a person's physical activity dose by 38% to 43% for running relative to the distance-based estimates. This estimate would appear to correspond well to the 44% greater energy expenditure calculated for time-based questionnaires than activity monitors [32]. Body weight will show the strongest association with the calculation that is closest to the objective measurement, which would be the distance-derived calculations. This suggests that subjects who meet the guideline activity levels by the time-derived estimate will actually fall short of the required dose by 28% to 31%. Because running is among the more common vigorous exercises [16], simple improvements their prescriptions could substantially improve efforts to reduce unhealthy weight through physical activity promotion. Our analyses suggest that this could be achieved by a simple reformulation of the guidelines to specify running targets (and walking targets) by distance rather than time.

### Caveats

An inherent limitation of cross-sectional analyses is the uncertainty of whether physical activity preceded body weight or whether the converse occurred. However, elsewhere we have shown that increases in BMI, body circumferences, and obesity were inversely related to baseline running distance when followed prospectively for seven years [11]. Moreover, other longitudinal data show that follow-up physical activity, rather than baseline activity, was the strongest predictor of weight gain during follow-up [12]. The recommended doses of physical activity for maintaining healthy weight by the Institute of Medicine were, in fact, themselves derived primarily from cross-sectional data of BMI and energy expenditure from doubly labeled water [5], [8]. Leaner individuals could self-select to run longer distances than to perform higher doses of other exercise. Although pre-exercise BMI accounts for 58% of the association between vigorous physical activity levels and BMI in women, it accounts for only a quarter of the association between vigorous physical activity levels and BMI in men [33]. In addition, the generalizability of the results may be affected by the initial recruitment of runners through footrace events and subscription lists to running oriented publications. This strategy was pursued in order to include higher exercise doses than represented in other population studies. However, we believe that the biological processes that relate running to body weight would not dramatically differ between the current sample and a less selected population, and the same bias would apply to all participants. We acknowledge that detailed dietary data, sleep, sedentary behaviors such as sitting, and other variables that could have affected body weight were not collected [12]. In particular, total calorie intake, a major determinant of BMI and body circumferences, was not obtained for the sample. Finally, we note that the purported tendency for overweight individuals to overestimate their physical activity [34] would diminish their inverse relationship, and thus could not explain the associations of Tables 2,3, and 5.

The current analyses also compared the relationship of METhr/d run to body weight using two different methods for calculating METhr/d run, one time-based and the other distance-based. Presumably any tendency for heavier runners to exercise less would affect both metrics, as well as other physical activities.

Finally, we note that the results of Tables 2 and 3 are apropos to the health consequences of greater body weight even though the majority of the runners (57.4% of men, 85.7% of women) were ostensibly healthy weight. Specifically, we have shown that greater BMI and larger waist circumference increase the risks for CHD even among normal-weight runners [35].

### Conclusions

Effective public health policies are required to address the impending obesity epidemic. The efficacy of the current physical activity guidelines to prevent obesity remains to be determined. While 69% of men and 60% of women report meeting the guideline activity levels [36], objective physical activity measurements suggest that the actual percentages may be less than 5% of adults [37]. There is even evidence to suggest that promotional messages to encourage exercise may actually increase food consumption [38]. Our analyses suggest that distance-based running prescriptions may provide greater health benefits than time-based prescriptions of other exercise. These results are consistent with those in walkers, which showed that distance walked to be a superior metric for relating the its energy expenditure to total and regional adiposity than its time-based calculation [39]. Additional longitudinal studies and randomized clinical trials are required to verify these results prospectively.

## Methods

### Ethics statement

The study protocol was reviewed and approved by the Human Subjects Committee at Lawrence Berkeley National Laboratory for the protection of human subjects, and all subjects provided a signed statement of informed consent.

The current analyses are based on a resurvey of the National Runners' Health Study [9], [10] who were recontacted in 2006 and who completed a four page survey on running history (average weekly mileage over the preceding 5 years, minutes required to run a mile, frequency of runs per week >10 min, longest usual run), height, current weight and circumferences of the chest, waist, and hips, diet (vegetarianism and the current weekly intakes of alcohol, red meat, fish, fruit), current and past cigarette use, and history of diseases. Intakes of meat, fish and fruit were based on the questions “During an average week, how many servings of beef, lamb, or pork do you eat”, “…serving of fish do you eat”, and “…pieces of fruit do you eat”. Alcohol intake was estimated from the corresponding questions for 4-oz (112 mL) glasses of wine, 12-oz (336 mL) bottles of beer, and mixed drinks and liqueurs. Alcohol was computed as 10.8 g/4-oz glass of wine, 13.2 g/12-oz bottle of beer, and 15.1 g/mixed drink. Education was solicited by requesting the participant provide “years of education (examples: HS = 12; BS or BA  = 16; MS or MA  = 18; PhD or MD  = 20)”.

Height and weight were determined by asking the participant, “What is your current height (in inches, without shoes)?” and, “What is your current weight (pre-pregnancy weight if pregnant)?” BMI was calculated as weight in kilograms divided by the square of height in meters. Self-reported waist, hip, and chest circumferences were elicited by the question, “Please provide, to the best of your ability, your body circumference in inches: waist___, hip___, and chest___,” without further instruction. Elsewhere, we have reported the strong correlations between self-reported and clinically measured heights (r = 0.96) and weights (r = 0.96) [40]. Self-reported waist, hip and chest circumferences were somewhat less precise, as indicated by their correlations with reported circumferences on a second questionnaire (r = 0.84, r = 0.79, r = 0.93, respectively) and with their clinical measurements (r = 0.68, r = 0.63, r = 0.77, respectively) [40]. Participants were also asked to report their body circumferences at age 18. Body mass index (BMI) between 18.5 and 24.9 kg/m^2^ was classified as healthy weight, between 25.0 and 29.9 kg/m^2^ as overweight, and 30 kg/m^2^ and above as obese [41]. Waist circumferences ≥102 cm in men and ≥88 cm in women were classified as abdominal obesity [41].

Running distance during the current year was reported in miles run per week, which was converted to kilometers per day. Previously, we reported strong correlations between repeated questionnaires for self-reported running distances (r = 0.89) [39]. In addition, the questionnaires asked “On average, how many hours per week do you spend running ___, walking ____, swimming ____, cycling ____, other exercise (describe in detail) ____.”, and “During your usual run, how many minutes does it take to run one mile?”.

Time based calculations of METhr/d of total, vigorous, moderate, and light exercise were calculated as the product of the average number hours per day spent on each activity and the estimated energy expenditure for the activity as listed in the 2000 compendium of physical activities [7]. Missing exercise durations were estimated as the average time spent at the activity from those that provided these data. The compendium gives the MET expenditures for running as 8 METs (12 min/mi), 9 METs (11.5 min/mi), 10 METs (10 min/mi), 11 METs (9 min/mi), 11.5 METs (8.5 min/mi), 12.5 METs (8 min/mi), 13.5 METs (7.5 min/mi), 14 METs (7 min/mi), 15 METs (6.5 min/mi), 16 METs (6 min/mi), and 18 METs (5.5 min/mi) [7]. The MET values provided in the compendium translate into an exercise dose that is solely a function of distance (1.02 kcal/kg or MET•hours per km, Figure 1). Time-based calculation of METhr/d run was computed by converting the reported time into distance (i.e., hours*kmph), which was then multiplied by 1.02 MET•hours per km.

### Statistical analyses

Statistical analyses were performed using JMP (SAS institute, Cary NC, version 5.1) and Stata (StataCorp LP, College Station TX, version 11). Table 1 presents means±SD for all variables assessed; all other statistics are expressed as mean±SE or coefficients±SE except where noted. Least-squares regression was used to estimate the relationships of BMI and body circumferences to METhr/d of running and other exercise. Logistic regression analyses were used to compute the odds for obesity (BMI>30 kg/m^2^), and abdominal obesity per METhr/d. Covariates included adjustments for age (age and age^2^), education, current smoking status, and intakes of meat, fruit, and alcohol. As these data are observational and cross-sectional, they cannot prove causality. The use of the terminology “increasing METhr/d” in reference to the independent variable and “decreasing BMI or body circumferences” in reference to the dependent variables pertain only to their mathematical functional relationship and is not intended to imply a causal relationship.

Two different tests were used to assess whether the distance-based calculation of METhr/d of running (Running_Distance_) differed from its traditional time-based calculation (Running_Time_) in their effect on BMI and body circumferences. Both use a model that simultaneously includes separate regression terms for each calculations of METhr/d for running: 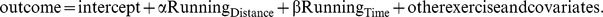

1. The standard test of significance for whether adding the distance-based estimate significantly improves the model over one that includes only the time-based estimate (i.e., α = 0), and correspondingly, whether adding the time-based estimate significantly improves the model over one that includes only the distance-based estimate (i.e., β = 0). 2) Direct comparison of the equivalence of the coefficients of the distance-based and time-based calculations via contrasts (i.e., α = β, equivalent to α-β = 0).
2. Similarly, the exchangeability premise, i.e., whether METhr/d from running differs from those of other exercise, was tested using linear contrasts to assess the significance of α = β in the equation: 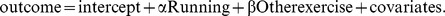

## References

[1] Pate RR, Pratt M, Blair SN, Haskell WL, Macera CA (1995). Physical activity and public health. A recommendation from the Centers for Disease Control and Prevention and the American College of Sports Medicine.. JAMA.

[2] US Department of Health and Human Services (1996). Physical Activity and Health: A Report of the Surgeon General. Atlanta (GA): US Department of Health and Human Services, Centers for Disease Control and Prevention, National Center for Chronic Disease Prevention and Health Promotion. 1999 278 p.. http://www.cdc.gov/nccdphp/sgr/pdf/sgrfull.pdf.

[3] Haskell WL, Lee IM, Pate RR, Powell KE, Blair SN (2007). Physical activity and public health: updated recommendation for adults from the American College of Sports Medicine and the American Heart Association.. Med Sci Sports Exerc.

[4] US Department of Health and Human Services (2008). Physical Activity Guidelines for Americans. Washington (DC): ODPHP Publication No. U0036. 2008. 61 p.. http://www.health.gov/paguidelines/pdf/paguide.pdf.

[5] Institute of Medicine (2005). Dietary Reference Intakes for Energy, Carbohydrate, Fiber, Fat, Fatty Acids, Cholesterol, Protein, and Amino Acids (Macronutrients). Washington DC: The National Academies Press.. (ISBN.

[6] U.S. Department of Agriculture and U.S. Department of Health and Human Services (2010). Dietary Guidelines for Americans, 2010.. 7th Edition, Washington, DC: U.S. Government Printing Office, December 2010.

[7] Ainsworth BE, Haskell WL, Whitt MC, Irwin ML, Swartz AM (2000). Compendium of physical activities: an update of activity codes and MET intensities. Med Sci Sports Exerc.. 32.

[8] Donnelly JE, Blair SN, Jakicic JM, Manore MM, Rankin JW (2009). Appropriate physical activity intervention strategies for weight loss and prevention of weight regain for adults. Med Sci Sports Exerc..

[9] Williams PT, Satariano WA (2005). Relationships of age and weekly running distance to BMI and circumferences in 41,582 physically active women. Obes Res..

[10] Williams PT, Pate RR (2005). Cross-sectional relationships of exercise and age to adiposity in 60,617 male runners. Med Sci Sports Exerc..

[11] Williams PT (2007). Maintaining vigorous activity attenuates 7-yr weight gain in 8340 runners. Med Sci Sports Exerc..

[12] Summerbell CD, Douthwaite W, Whittaker V, Ells LJ, Hillier F (2009). The association between diet and physical activity and subsequent excess weight gain and obesity assessed at 5 years of age or older: a systematic review of the epidemiological evidence. Int J Obes (Lond)..

[13] Goran MI, Treuth MS (2001). Energy expenditure, physical activity, and obesity in children.. Pediatr Clin North Am.

[14] O'Donovan G, Blazevich AJ, Boreham C, Cooper AR, Crank H (2010). The ABC of Physical Activity for Health: a consensus statement from the British Association of Sport and Exercise Sciences. J Sports Sci..

[15] Tremblay A, Després JP, Leblanc C, Craig CL, Ferris B (1990). Effect of intensity of physical activity on body fatness and fat distribution. Am J Clin Nutr..

[16] Tudor-Locke C, Johnson WD, Katzmarzyk PT (2010). Frequently reported activities by intensity for U.S. adults: the American Time Use Survey. Am J Prev Med..

[17] Andrade AM, Coutinho SR, Silva MN, Mata J, Vieira PN (2010). The effect of physical activity on weight loss is mediated by eating self-regulation. Patient Educ Couns..

[18] Martins C, Morgan L, Truby H (2008). A review of the effects of exercise on appetite regulation: an obesity perspective.. Int J Obes.

[19] Pendleton VR, Goodrick GK, Poston WS, Reeves RS, Foreyt JP (2002). Exercise augments the effects of cognitive-behavioral therapy in the treatment of binge eating.. Int J Eat Disord.

[20] Baker CW, Brownell KD, Bouchard C (2000). Physical activity and maintenance of weight loss: physiological and psychological mechanisms..

[21] King NA, Hopkins M, Caudwell P, Stubbs RJ, Blundell JE (2008). Individual variability following 12 weeks of supervised exercise: identification and characterization of compensation for exercise-induced weight loss.. Int J Obes.

[22] O'Rahilly S, Farooqi IS (2008). Human obesity: a heritable neurobehavioral disorder that is highly sensitive to environmental conditions. Diabetes..

[23] Martins C, Morgan LM, Bloom SR, Robertson MD (2007). Effects of exercise on gut peptides, energy intake and appetite.. J Endocrinol.

[24] Broom DR, Batterham RL, King JA, Stensel DJ (2009). Influence of resistance and aerobic exercise on hunger, circulating levels of acylated ghrelin, and peptide YY in healthy males.. Am J Physiol Regul Integr Comp Physiol.

[25] Martins C, Truby H, Morgan LM (2007). Short-term appetite control in response to a 6-week exercise programme in sedentary volunteers.. Br J Nutr.

[26] Catenacci VA, Wyatt HR (2007). The role of physical activity in producing and maintaining weight loss.. Nat Clin Pract Endocrinol Metab.

[27] Jakicic JM, Marcus BH, Lang W, Janney C (2008). Effect of exercise on 24-month weight loss maintenance in overweight women.. Arch Intern Med.

[28] Willer CJ, Speliotes EK, Loos RJ, Li S, Lindgren CM (2009). Six new loci associated with body mass index highlight a neuronal influence on body weight regulation. Nat Genet..

[29] Yoshioka M, Doucet E, St-Pierre S, Alméras N, Richard D (2001). Impact of high-intensity exercise on energy expenditure, lipid oxidation and body fatness.. Int J Obes Relat Metab Disord.

[30] Ratcliff L, Gropper SS, White BD, Shannon DM, Huggins KW (2011). The influence of habitual exercise training and meal form on diet-induced thermogenesis in college-age men. Int J Sport Nutr Exerc Metab..

[31] Lennon D, Nagle F, Stratman F, Shrago E, Dennis S (l985) Diet and exercise training effects on resting metabolic rate.. Int J Obes.

[32] Prince SA, Adamo KB, Hamel ME, Hardt J, Gorber SC (2008). A comparison of direct versus self-report measures for assessing physical activity in adults: a systematic review. Int J Behav Nutr Phys Act..

[33] Williams PT (2008). Self-selection accounts for inverse association between weight and cardiorespiratory fitness. Obesity (Silver Spring)..

[34] Lichtman SW, Pisarska K, Berman ER, Pestone M, Dowling H (1992). Discrepancy between self-reported and actual caloric intake and exercise in obese subjects. New Engl J Med..

[35] Williams PT, Hoffman KM (2009). Optimal body weight for the prevention of coronary heart disease in normal-weight physically active men. Obesity (Silver Spring)..

[36] The Centers for Disease Control and Prevention (CDC) (2010). Prevalence of self reported physically active adults–United States, 2007.. MMWR Morb Mortal Wkly Rep 2008.

[37] Troiano RP, Berrigan D, Dodd KW, Mâsse LC, Tilert T (2008). Physical activity in the United States measured by accelerometer. Med Sci Sports Exerc..

[38] Albarracin D, Wang W, Leeper J (2009). Immediate increase in food intake following exercise messages. Obesity (Silver Spring)..

[39] Williams PT (2012). Advantage of distance- versus time-based estimates of walking in predicting adiposity. Med Sci Sports Exerc.. In press.

[40] Williams PT (2004). Vigorous exercise and the population distribution of body weight. Int J Obes Relat Metab Disord..

[41] Clinical Guidelines on the Identification, Evaluation, and Treatment of Overweight, Obesity in Adults: The Evidence Report: National Institutes of Health (1998). Obes Res.

